# TRENDS IN SPASTICITY-REDUCING SURGERY AND BOTULINUM TOXIN TREATMENT FOR POST-STROKE SPASTICITY: A REGISTER STUDY ON 6,258 PATIENTS IN SWEDEN, 2010–2021

**DOI:** 10.2340/jrm.v57.42684

**Published:** 2025-03-18

**Authors:** Marcus SAGERFORS, Izabela BLASZCZYK, Anette CHEMNITZ, Helena JOHANSSON, Joakim STRÖMBERG

**Affiliations:** 1 Department of Orthopedics and Hand Surgery, Faculty of Medicine and Health, Örebro University, Örebro; 2 Department of Diagnostic and Intervention, Hand and Plastic Surgery, Umeå University, Umeå; 3 Hand Center Malmö, Atleva Specialist Care, Malmö; 4 Sahlgrenska Osteoporosis Centre, Department of Internal Medicine and Clinical Nutrition, Institute of Medicine, University of Gothenburg, Gothenburg; 5 University of Gothenburg, Sahlgrenska Academy, Institution of Clinical Sciences, Department of Hand Surgery and Department of Surgery and Orthopaedics, Alingsås lasarett, Alingsås, Sweden

**Keywords:** botulinum toxin, spasticity, stroke, tendon transfer, extremity surgery

## Abstract

Spasticity is characterized by increased muscle tone, which can result in pain, contractures, impaired hygiene, and deformities. Stroke is a leading cause of paresis, and nearly 40% of stroke patients will develop spasticity.

**Objective:**

To assess trends in upper and lower extremity spasticity-reducing surgery and botulinum toxin A (BoNT-A) treatment.

**Design:**

A national cohort register study.

**Methods:**

Upper and lower extremity spasticity-reducing surgery and BoNT-A treatment in Swedish stroke patients over a 12-year period was assessed using the National Patient Register.

**Results:**

A total of 6,258 patients were treated during this period; their mean age was 58, and the majority were male. In both upper and lower extremities, tenotomy was the most common surgical procedure, followed by tendon lengthening. The need for BoNT-A injections was significantly reduced after surgery compared with before surgery. The total number of BoNT-A treatments increased during the study period, and ultrasound guidance of injections became more common.

**Conclusion:**

The frequency of BoNT-A treatments was significantly reduced in patients who underwent surgery. Even though no causative association can be established due to the nature of these registry data, this may indicate that surgery reduces the need for further BoNT-A treatments.

## LAY ABSTRACT

Stroke is a common cause for paresis. Many stroke patients develop spasticity with increased muscle tone resulting in pain, contractures, and deformities. Spasticity-related symptoms can be treated with stretching, splints, baclofen, botulinum toxin A (BoNT-A), and surgery. This study explores trends in BoNT-A treatment and spasticity-reducing surgery in the upper and lower extremities by using data from the National Patient Register in Sweden during a 12-year period. The findings indicate that a total of 6,258 patients were treated, the majority of whom were male; their mean age was 58. The 2 most common surgical procedures were tenotomy (sectioning of the tendon) and tendon lengthening. The frequency of BoNT-A injections was significantly reduced after surgery compared with before surgery. Although no causative association can be established due to the nature of this study, the findings may indicate that surgery reduces the need for further BoNT-A treatments.

Spasticity is a motor disorder characterized by an increase in muscle tone and hyperexcitable stretch reflexes. In addition to impaired muscle function, it may cause pain, contractures, impaired hygiene, and deformities (1). Several conditions can result in spasticity, such as cerebrovascular incidents (stroke), cerebral palsy, and traumatic brain injury. Stroke is the leading cause of paresis in the United States, and 38% of stroke patients will develop spasticity (2).

Spasticity-related symptoms in the upper and lower extremities may be alleviated by non-surgical treatment (3) such as stretching, splints, oral or intrathecal baclofen therapy, and botulinum toxin A (BoNT-A) treatment. Surgical treatment of spasticity can include tenotomy or tendon lengthening, joint stabilization, and recently peripheral nerve surgery using selective or hyperselective neurectomy (4, 5). A randomized controlled trial found that tendon transfer surgery was more effective than serial BoNT-A treatments for paediatric patients with spastic unilateral cerebral palsy (6).

Surgery clearly has a role in the management of spasticity (7), but many patients are referred late or not at all (8). Despite the efficacy of surgical interventions for upper and lower extremity spasticity, there is a substantial underutilization of surgery in patients with spasticity from stroke or traumatic brain injury (9). The primary aim of this register study was to assess spasticity-reducing surgical interventions and BoNT-A treatments in Swedish patients with spasticity in the extremities over a 12-year period (2010–2021). Secondary aims were to assess whether spasticity-reducing surgery could reduce the need for BoNT-A treatments in adult patients, and to examine any regional differences within Sweden.

## MATERIALS AND METHODS

Ethical approval was granted by the Swedish Ethical Review Authority (No 2022-01998-01). Data for the study were drawn from the National Patient Register (NPR), which is administered by the National Board of Health and Welfare (https://www.socialstyrelsen.se/en/). After review and consent by their own data safety committee, the Swedish authorities delivered pseudonymized data using anonymized personal identification numbers for the linking of data.

Discharge diagnoses from all hospitals and outpatient clinics in Sweden are recorded in the NPR according to the International Classification of Diseases and Causes of Death, 10^th^ version (ICD-10), and clinical and surgical procedures are recorded in accordance with the Swedish Classification of Care Procedures. For the present study, we identified patients with ICD-10 diagnosis codes I69.0–I69.9 (stroke), G81.1 (spastic hemiparesis), or G82.4 (spastic tetraparesis) during the period 2010–2021, and extracted data on BoNT-A treatments and spasticity-reducing surgery in the upper or lower extremities for these individuals during the same time period.

### Statistical analyses

Descriptive statistics were calculated as mean values and standard deviations, or as counts and percentages for categorical variables. The difference in number of BoNT-A treatments per year before and after surgery was assessed with the non-parametric Wilcoxon signed-rank test. A two-sided *p*-value of < 0.05 was considered statistically significant. Statistical analyses were performed using version 26 of SPSS (IBM Corp, Armonk, NY, USA).

## RESULTS

**Fig. 1:**
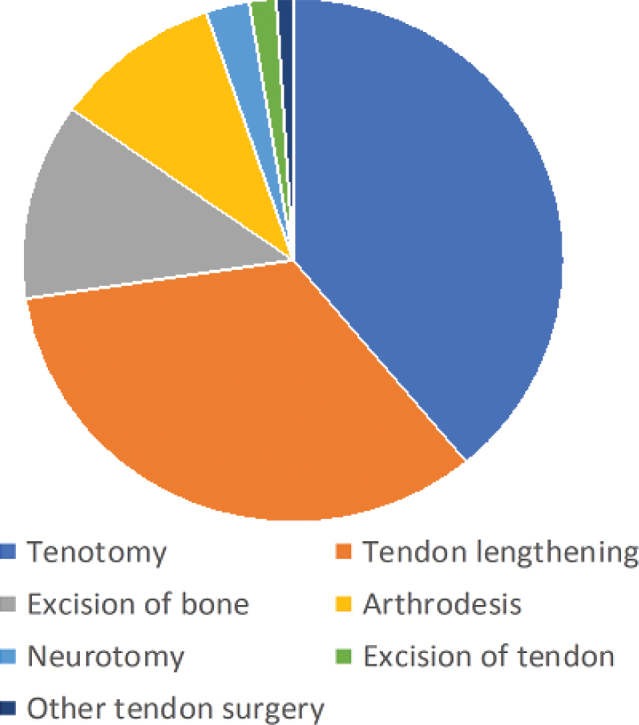
Frequency of surgical procedures for spasticity in the upper extremity.

**Fig. 2:**
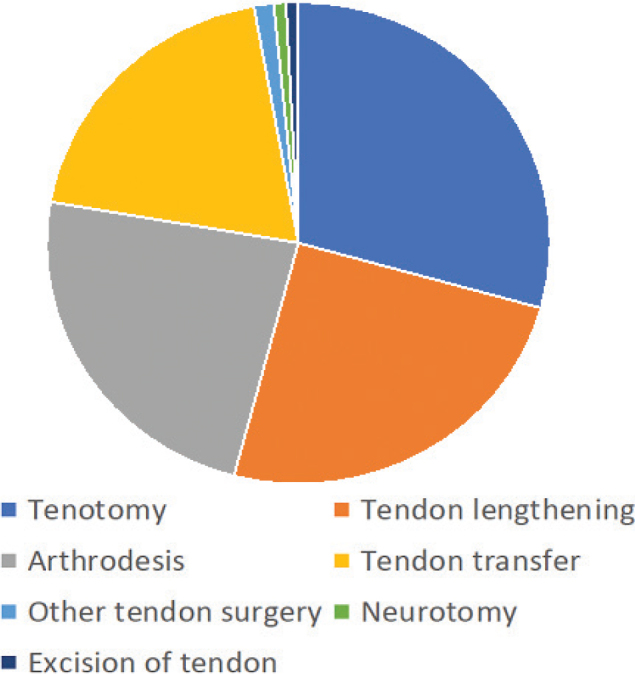
Frequency of surgical procedures for spasticity in the lower extremity.

This register study identified a total of 110,419 visits by 6,258 patients between 2010 and 2021. The patients’ mean age was 58, and 59% of them were male. All had a diagnosis of stroke/late effects of cerebrovascular disease, sometimes in combination with dementia or Parkinson’s disease. A total of 1,223 patients (19.5%) had undergone upper or lower spasticity-reducing surgery (Table I). The most common surgical intervention for the upper extremity was tenotomy, followed by tendon lengthening. Tendon transfers were uncommon (Fig. 1). Neurectomy in the upper extremity was most commonly performed on the ulnar nerve. The most common surgery for the lower extremity was again tenotomy, followed by tendon lengthening (Fig. 2).

**Table I:** Demographics, diagnoses, and surgical procedures in patients treated for post-stroke spasticity in Sweden between 2010 and 2021

Total number of treatments (non-surgical and surgical)	110,419
Total number of patients	6,258
Age in years, mean (SD)	58 (15.9)
Gender, *n* (%)	
Male	3,669 (59%)
Female	2,589 (41%)
Diagnosis, *n* (%)	
Late effects of cerebrovascular disease	5,925 (95%)
Dementia + late effects of cerebrovascular disease	232 (4%)
Parkinson’s disease + late effects of cerebrovascular disease	83 (1%)
Dementia + Parkinson’s disease + late effects of cerebrovascular disease	18 (0.3%)
Surgical procedures	
Total number	1,223
Percentage of all treatments	19.5%
Upper extremity	595
Lower extremity	610
Upper and lower extremity	18

Patients who underwent spasticity-reducing surgery of the upper or lower extremity and who had BoNT-A treatments both before and after surgery (*n* = 865) had a significantly lower number of BoNT-A treatments per year after surgery (0.9 injections/year) compared with before surgery (1.5 injections/year) (*p* < 0.001).

**Fig. 3:**
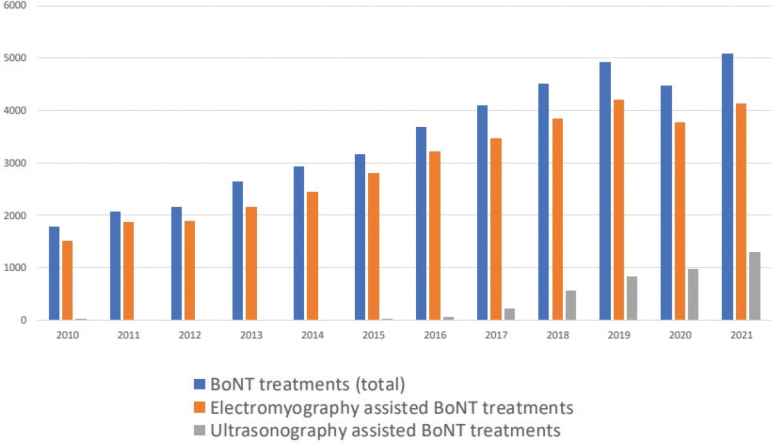
Total number of botulinum toxin A (BoNT-A) treatments 2010–2021.

**Fig. 4:**
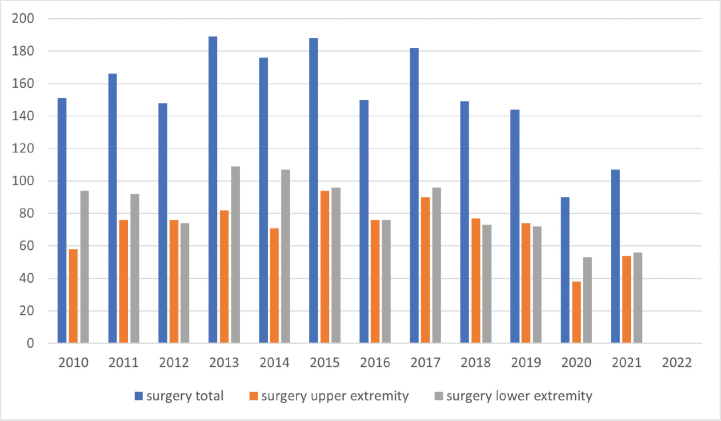
Frequency of spasticity-reducing surgical procedures in Sweden, 2010–2021, divided by year.

**Fig. 5:**
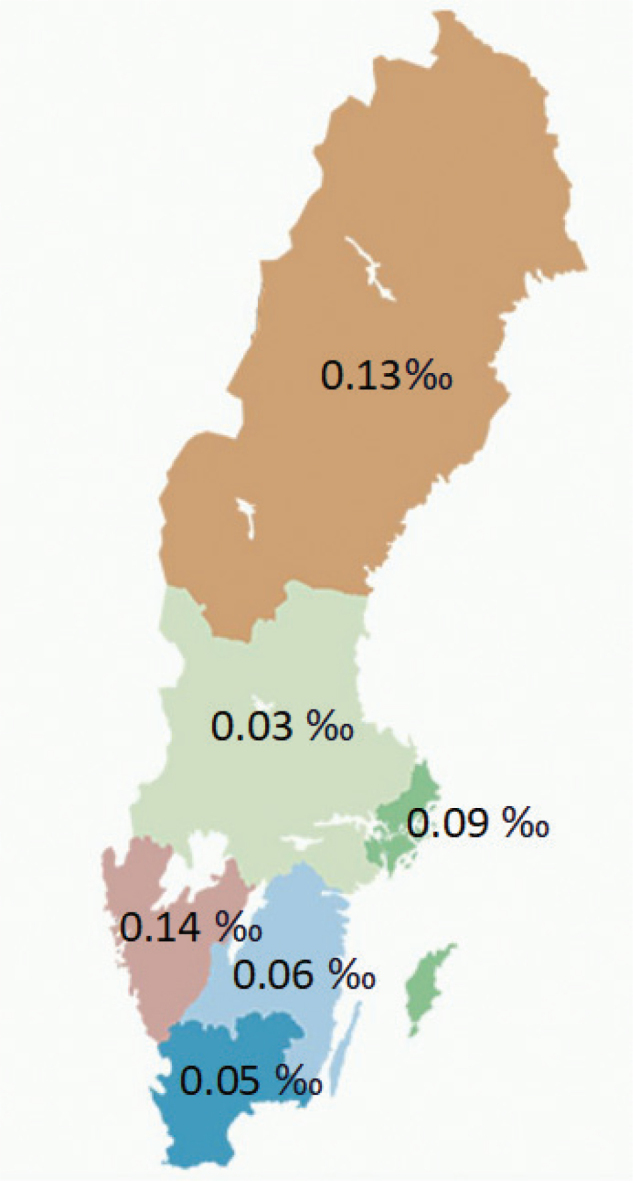
Frequency of spasticity-reducing surgical procedures in Sweden, 2010–2021, divided by region.

The number of BoNT-A treatments increased substantially during the study period, from almost 2,000 per year in 2010 to 5,000 in 2021. Most treatments were carried out using electromyography. Greater use of ultrasonography-assisted BoNT-A treatments was noted in the latter part of the period, increasing from 1% in 2015 to 30% in 2021 (Fig. 3). The utilization of spasticity-reducing surgery for the upper and lower extremities did not increase over time (Fig. 4). There were substantial regional differences regarding the frequency of spasticity-reducing surgery, varying from 0.03‰ to 0.14‰ in relation to the regional population (Fig. 5).

## DISCUSSION

The main finding in this study was that patients who were treated surgically received fewer BoNT-A treatments after these procedures. Even though no causative association can be established due to the nature of these registry data, this may indicate that surgery reduces the need for further BoNT-A treatments. The treatment of spasticity in the extremities by using BoNT-A or spasticity-reducing surgery is symptomatic, not causal. At present, there is no effective causal treatment method of spasticity resulting from injury to the central nervous system. The spasticity-reducing effect of BoNT-A treatment is temporary, so the treatment needs to be repeated every 3–6 months (10). In contrast, spasticity-reducing surgery has been shown to provide long-lasting effects for up to 6 years (11).

Surgical procedures to reduce spasticity symptoms are aimed at reducing the consequences of spasticity, as the primary injury to the central nervous system is not surgically curable. For the spastic extremity, the aim of surgery is not only to reduce the spasticity by means of neurectomy, but also to correct any fixed contractures by performing tenotomy, muscle release, tendon lengthening, and tendon transfer, or by using arthrodesis to stabilize a joint in a better and often less painful and more functional position. In the upper extremity, surgical goals often include facilitating hygiene and improving posture, function, and pain (6, 12).

Patients with hemiplegia following stroke often exhibit malposition of the distal lower limb in terms of equinus, varus, and clawing toes (13). Procedures mainly target nerves (neurotomy) and tendons (lengthening, transfer, tenotomy), but joint arthrodeses are also an option. These procedures can reduce impairments (spasticity, range of motion, and foot position) and improve gait and walking function (14). In addition, surgical interventions for a spastic upper extremity can improve lower extremity kinematics in adults with spasticity (15), and have long-lasting effects (11).

There are data indicating a considerable underutilization of reconstructive surgery in patients with spasticity from a stroke or traumatic brain injury (9). Likely reasons for this underutilization are the wide variety of procedures available and the frequent lack of a clear consensus on treatment, making it hard to provide an individual work-up and surgical intervention (16). We saw the same pattern in our cohort.

The stroke incidence in Sweden is decreasing; the current prevalence is 1,518 per 100,000 inhabitants (1,754 for men and 1,281 for women) (17). Children in Sweden with cerebral palsy undergo an annual follow-up measuring the range of motion of their upper and lower extremities, and occupational therapists have instructions to notify the treating physician if they observe any deterioration (18). This setup and follow-up programme is not available for adult patients with spasticity, but could hypothetically give a better chance of identifying problems early and hence preventing consequences such as joint contractures and skin breakdown in the palm. A recent systematic review found that the incidence of post-stroke spasticity was significantly higher in stroke patients with paresis. Thus, patients with moderate to severe paresis and sensory disorder should be closely followed up (19).

A majority of the patients in our cohort were male, which is in line with the population of stroke patients in Sweden (17). However, the mean age in our cohort was 58 years, which is substantially lower than the mean age among patients living with a stroke diagnosis in Sweden: 74 years for men and 75 years for women (17). We speculate that younger stroke patients are more likely than their older counterparts to be referred for interventions such as BoNT-A treatments and surgery. It therefore seems likely that the underutilization of spasticity-reducing treatment for those who have had a stroke is even larger in older patients. This is supported by the findings by Levy et al., who found that the proportion of stroke patients treated with BoNT-A decreases substantially with increasing age (20).

Tenotomy was the most common procedure in our material, followed by tendon lengthening. However, the available data did not allow us to see which types of tenotomy or lengthening were performed. Common procedures described for the upper extremity include lengthening of the biceps, brachialis, pronator teres, and wrist flexors (7, 21). Our data did not include any instances of tendon transfer being performed in stroke patients. The reason for this remains unknown; but in our experience, these procedures are more often performed in patients with relatively high hand/arm function. We speculate that stroke patients may have low voluntary control of the affected extremity, resulting in tenotomies and lengthenings rather than tendon transfers. For neurectomies, the ulnar nerve was the nerve most commonly operated on. The exact level of the neurectomy was not recorded in our dataset, but these procedures most likely represent neurectomy of the motor branch of the ulnar nerve to reduce the intrinsic plus position of the fingers of the spastic hand (22).

The frequency of BoNT-A treatments using ultrasonography guidance increased considerably during the study period. A previous study has indicated that electromyography and ultrasonography have similar efficacy, but the number of patients was small and ultrasonography is probably more time efficient (23). We saw no increase in the frequency of surgical procedures.

All patients in the cohort had a diagnosis of stroke, sometimes in combination with other diagnoses such as Parkinson’s and dementia. This finding probably reflects the fact that these diagnoses were made during the 12-year study period; for example, a patient with Parkinson’s may have had a stroke later in the study period. Furthermore, we found a regional difference in the frequency of spasticity-reducing surgery among the Swedish regions, even after correction for the number of inhabitants per region. As it is unlikely that the incidence of stroke varies across the regions, this may indicate inequality of access to care. This is supported by the findings of Forsmark et al., who found a marked variation in pharmacologic treatment of adult spasticity in Sweden. In addition, the findings also indicated an underuse of treatment and a need for general clinical guidelines (24).

One limitation of this study is that patient-reported outcome measures were not available. Collecting such measures in this group of patients can be challenging due to aphasia and dementia. Another limitation is that the patients were not randomized to treatment. On the other hand, the data reflect the treatment given to a large national cohort of patients over a 12-year period.

In conclusion, our findings indicate that upper and lower extremity surgical interventions to reduce spasticity symptoms can significantly reduce the frequency of BoNT-A injections. Undertreatment of upper and lower extremity spasticity in this patient group is likely. Further studies are warranted to clarify the role of spasticity surgery for stroke patients, preferably including prospective collection of patient-reported outcome measures.
